# Rhino facial zygomycosis: case report

**DOI:** 10.1590/S1679-45082014RC2579

**Published:** 2014

**Authors:** Juliana Miguita e Souza, Antonio José Sproesser, Alexandre Felippu, Florencia Barbero Fuks, Carlos Augusto Cardim de Oliveira

**Affiliations:** 1 Hospital Israelita Albert Einstein, São Paulo, SP, Brazil.

**Keywords:** Zygomycosis/therapy, Zygomycosi/diagnosis, Magnetic resonance imaging, Tomography, X-ray computed, Case reports

## Abstract

Zygomycosis is an invasive disease that affects both immunocompetent and immunocompromised, depending on the type of strain. This disease diagnosis is clinical and histopathological, and its treatment is based on antifungal therapy and surgical cleaning. This paper reports a case of a boy with invasive zygomycosis rinofacial who final treatment was successful after underwent antifungal and surgical therapies.

## INTRODUCTION

Zygomycosis is a rare fungal, severe, invasive and lethal disease with rapid progress. This disease is prominent in countries with warm and humid climate.^(1, 2)^ Its incidence in Brazil is unknown because of the scarcity of reports in the literature, but it is well known that several cases occur in north and northeast region of the country.^(3)^ There is a tendency in international literature, mainly in immunocompromised patients, of increase in the incidence of fungal invasive disease. An incidence of zygomycosis of 2-4% was found in autopsies of cancer patients both in Japan and the United States.^(3)^

The zygomycosis has several clinical presentations (cutaneous, pulmonary, systemic and gastrointestinal), however, the rhino-orbital-cerebral is the most common one.^(4, 5)^

Zygomycosis etiologic agents originate from *Mucorales* that primarily affects immunocompromised patients and base disease carriers, and from the *Entomophthorales* that is commonly found in immunocompetent individuals and rarely causes angioinvasive disease.^(1, 5)^

The diagnosis is based on a combination of mycologic and histopathological tests, and clinical presentation.^(6)^ The fungal infection could be determined by direct examination with potassium hydroxide (KOH) and culture on Sabouraud’s medium. The histopathological analysis shows nonseptate hyphae with branches at 90° angle, which usually does not invade tissues and blood vessels.^(7)^

Common symptoms include nasal congestion, nasal discharge and chronic sinusitis,^(8)^ however, the infection may also present fever, lethargy, headache, retro-orbital pain, sudden vision loss, proptosis, periorbital cellulitis, epistasis and seizure.^(9)^

At physical examination necrotic crusts can appear on the nasal septum, turbinates and palate. Initially, the orbital involvement can be show by proptosis and periorbital cellulitis with subsequent ophtalmoplegy and amaurosis.^(6)^ Later, the spread of the infection to the central nervous system can occur by the ethmoid bone and sphenoid sinus after bone destruction.

Treatment success depends on rapid diagnosis^(5)^ and the combined approach of surgical procedures and antifungal therapy.^(4)^ The amphotericin B is the drug of choice for initial treatment. There are studies reporting increase of up to 79% in patient survival after adoption of this drug. For the effective control of the disease, high doses of amphotericin B must be used. These doses range from 0.8 to 1.5mg/kg daily, which correspond to doses very close to nephrotoxic levels.^(9)^

The use of hematopoietic as stimulating factors and hyperbaric oxygen therapy may be beneficial, but few studies had established the efficacy of these procedures.^(10)^ There are also studies reporting success by the oral administration of potassium iodide.^(1, 6)^ Surgical debridement, which main goal is to remove as much devitalized tissue as possible, in addition to establish adequate sinus drainage, has a significant impact on the disease morbidity and mortality.^(1)^

Prevention of the disease consists in environmental control, avoidance or reduction of direct contact with fungal propagules, which are found in plants, flowers and house dust.^(7, 10)^

Poor prognosis factors are delayed initiation of treatment, intracranial (hemiplegia and hemiparesis), palate or orbital involvement as well as bilateral facial sinus involvement, and facial necrosis.^(7)^

This paper reports a case of immunocompetent patient with late diagnosis and who initially had unfavorable clinical evolution. Treatments were based on the experience of the responsible physician and case reports previously published in the literature. The result was total remission of the disease and no sequelae.

## CASE REPORT

**Figure 1:**
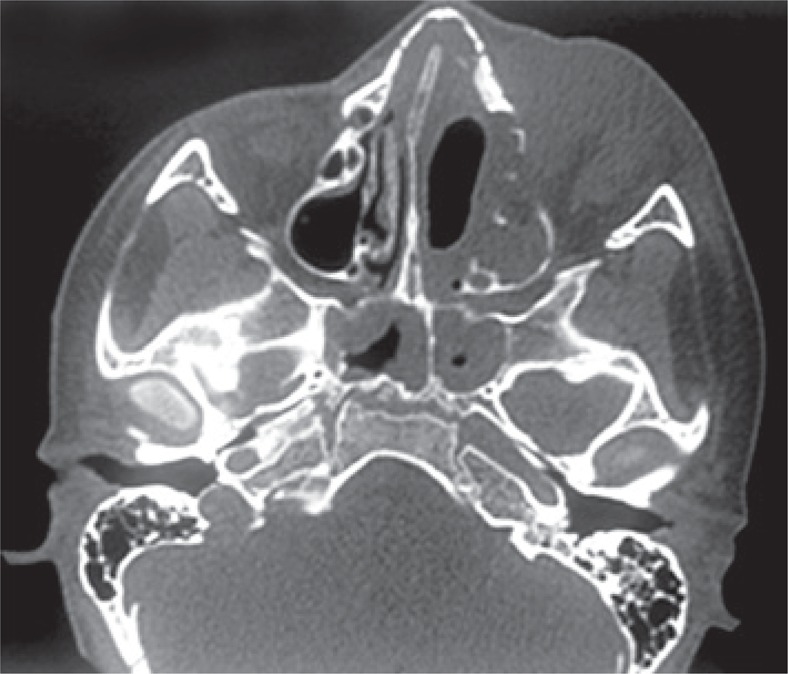
Computed tomography of the face sinuses. Signs of persistence of lesion in edges of surgical sites toward left pterygopalatine fossa, remaining antral cavities to the left and large orbital component also to the left, which infiltrated pre and post-septal plans and the lachrymal apparatus

**Figure 2:**
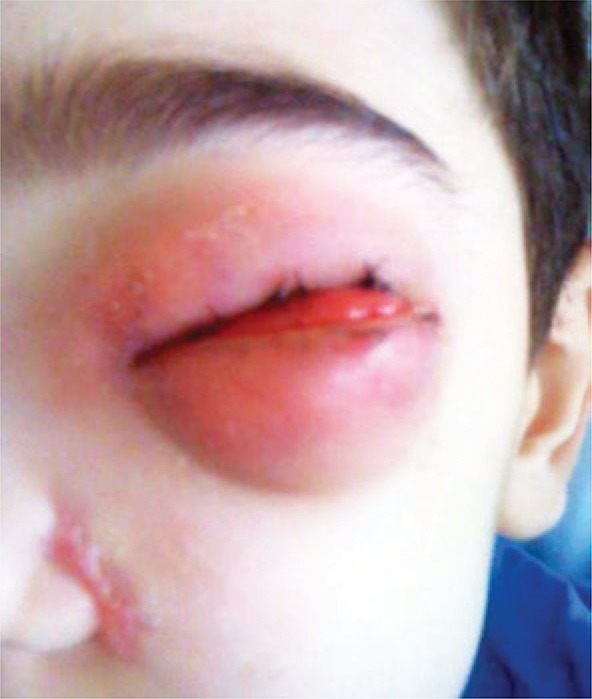
External involvement of facial mycosis

A 9-year-old boy from city of Belém (PA) was referred to the pediatric unit of the *Hospital Israelita Albert Einstein* (HIAE) in São Paulo, for treatment of rhino facial zygomycosis. He was a previously healthy and had a habit of smelling roses. In March 2010, the patient presented tumors inside his left nostril that increased progressively. The child was diagnosed with cellulitis and treated with antibiotics, topic and systemic corticoids, however, the lesions did not improve but got worse. The computed tomography of the skull revealed sinusopathy of left paranasal sinuses (Figure 1). Almost 3 months later, the patient was submitted to surgical cleaning of facial sinus along with resection of lesion on nasal wall. The histopathological exam showed zygomycosis. After two weeks, he become to present painful cellulitis in orbital region (Figure 2). Treatment was initiated with amphotericin B and caspofungin. Despite the introduction of medication, the disease continued to progress and the pain worsened, being also observed a decrease in ocular motility and proptosis. The magnetic resonance test showed periorbital invasion with probable contiguity to the skull base (Figure 3). With this clinical picture, the child was submitted again to two surgical cleanings, and the antifungal treatment was extended. Subsequently, the patient condition improved and lesions and periorbital edema reduced. The *Conidiobollus sp* was isolated from paranasal secrections culture.

**Figure:**
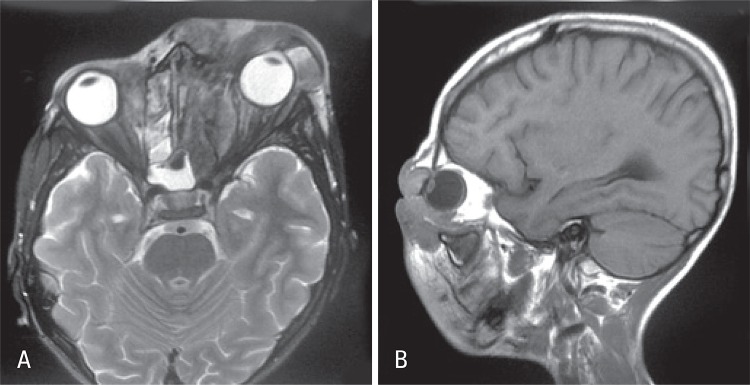
Figure 3 (A and B). Infiltrative lesion that affects the face and sinuses, especially the left orbital region, nose and oropharynx, with greater accumulation of secretions in the sinuses and slight increase in edema and/or inflammation at the base of the anterior cerebral fosse

After stabilization of patient’s condition, he returned to Belém to complete the treatment with amphotericin B. The appearance of side effects such as hypocalcemia, hyperthermia and body pain caused his hospitalization at intensive care unit for hemodynamic and hydroelectrolytic stability. After his discharge from intensive care unit, the treatment was replaced by oral antifungal posaconazole that caused a worsening in clinical picture.

**Figure 4:**
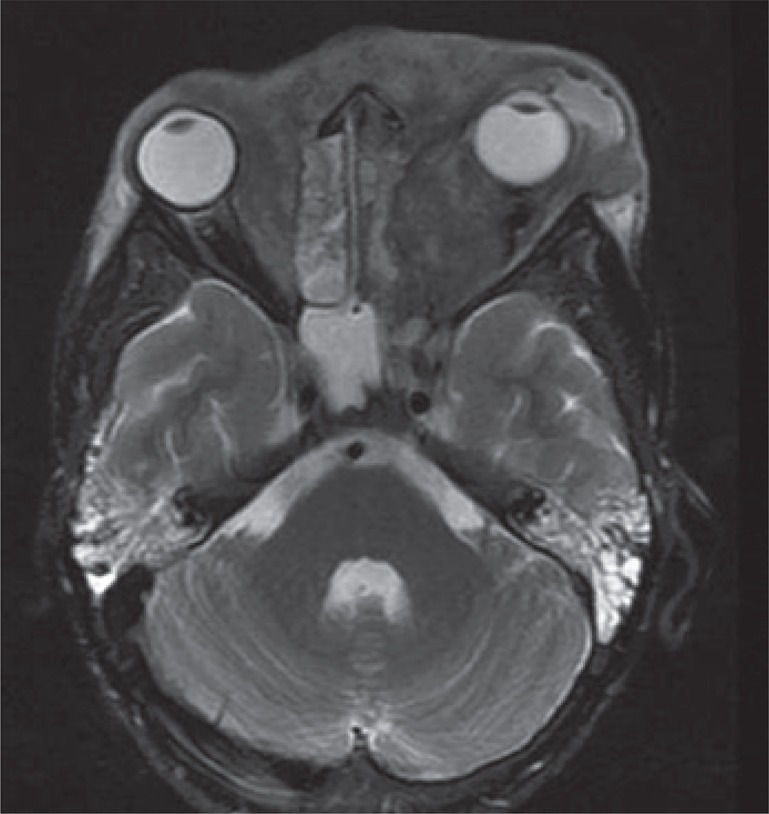
Thickening and enhancement involving bulb and olfactory nerves and the floor of the anterior cranial fossa. There is displacement of the base of the left frontal lobe and suggestive lesions of inflammation and/or edema in straight orbital`s frontal gyri at the same side

**Figure 5:**
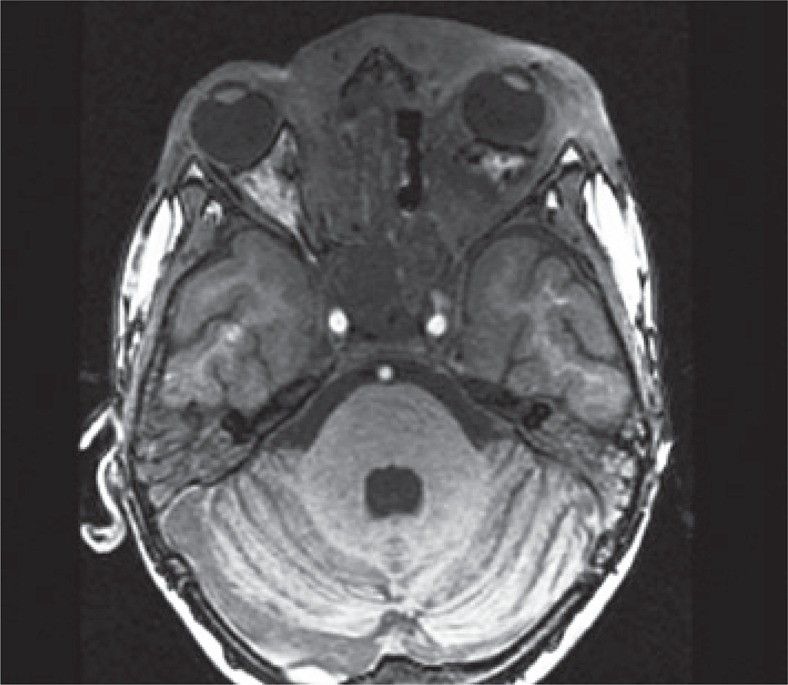
Decreased infiltrative lesion that affects the subcutaneous tissue, mainly in the left periorbital region and reduction of proptosis at the same side. Lesion reduction in nasopharynx, hypopharynx and sinuses, compared to previous images

The patient returned to our service in August 2010 and a new magnetic resonance exam of the skull showed progression of infiltrative lesions affecting the left hemiface, orbits and large extension of ethmoid and sphenoid sinus, besides affect the pachymeningeal (Figure 4). Because of clinical picture worsening, persistence fever and complications related to hospitalization a therapeutic scheme was used with amphotericin B, caspofungin, terbinafine, terbinafine, itraconazole, corticoids, acyclovir, erythropoietin, ceftriaxone, cefepime and ciprofloxacin at different times and also hyperbaric oxygenotherapy. Fifteen days after the last session of hyperbaric chamber, a magnetic resonance showed lesions but without signs of invasion of healthy tissues (Figure 5). After almost 2 months, the patients underwent another surgical cleaning and at that time there were improvement on obstructive symptoms and practically, the cure of the disease.

## DISCUSSION

The *Conid* *iobollus sp* is a saprophytic organism that grows on the floor and propagules in vegetation. This parasite often affect arthropods; human infection is accidental.^(10)^ Fungal infections can be acquired by inhalation or direct inoculation, being this latter the case of our patient.^(8)^

The most common presentation of the disease is an initial clinical picture with nasal lesion of rapid growing that provoke obstructive signs that, within few weeks, spread to periorbital region and cause edema, ptosis and proptosis.^(1)^

The late diagnosis, about 3 months, justifies the high morbidity of this disease and it can be explained by several types of differential diagnosis for the initial symptoms.^(2)^ In our case, the skin lesion was first treated as impetigo and other obstructive symptoms such as sinusitis. The lack of therapeutic response resulted in surgical cleaning and biopsy.^(3)^

It is important to mention that intravenous amphotericin B has been considered the treatment of choice.^(1, 6, 7)^ In our patient, this fact was well registered because in the period that he received this medication by the adequate route the response was more satisfactory. However for a completely adequate treatment it is necessary to be careful with drug administration in order to avoid side effects, which were also observed in the child in our report.

The hyperbaric oxygenotherapy might contributed to the improvement, although there is no evidences of its use for treatment of zygomycosis.^(10)^ The surgical cleaning is an essential part of the therapeutic strategy, because it removes as much infected material as possible and enables to observe the sinus drainage, which was observed in our case. For this reason, the use of surgical cleaning should be emphasized.^(7)^

## CONCLUSION

The zygomycosis is a severe fungal disease that can affect both immunocompetent and immunocompromised individuals.

The literature shows that rapid diagnosis and early adequate treatment are fundamental for the disease prognosis. The patient described in this case, although with little delay in the diagnosis, had a favorable progress due to treatments used.
