# Supplementary material for: Rhino facial zygomycosis: case report
Source: Einstein (Sao Paulo). 2014 Jul-Sep;12(3):347–50. doi: 10.1590/S1679-45082014RC2579 (PMC4872948; doi:10.1590/S1679-45082014RC2579)
Supplement: Translation

# Translation: Zigomicose rino facial: relato de caso

**Authors:** Juliana Miguita e Souza, Antonio José Sproesser, Alexandre Felippu, Florencia Barbero Fuks, Carlos Augusto Cardim de Oliveira

**Affiliations:** 1 Hospital Israelita Albert Einstein, São Paulo, SP, Brasil.

**Keywords:** Zigomicose/terapia, Zigomicose/diagnóstico, Imagem por ressonância magnética, Tomografia computadorizada por raios-x, Relatos de casos

## Abstract

A zigomicose é uma doença invasiva, que acomete tanto imunocompetentes como imunocomprometidos, dependendo do tipo da cepa. O diagnóstico é clínico e histopatológico, e o tratamento é baseado em antifúngico e em limpeza cirúrgica. O presente relato de caso é sobre um menino com zigomicose rinofacial invasiva com tratamento final bem-sucedido, após terapias antifúngicas e limpezas cirúrgicas.

## INTRODUÇÃO

Zigomicose é uma doença fúngica rara, grave e invasiva, de evolução rápida e fulminante, que predomina em países de clima úmido e quente.^(1, 2)^ A incidência dessa doença no Brasil é desconhecida, já que existem apenas poucos relatos de casos descritos na literatura, porém sabe-se que há um predomínio de casos nas Regiões Norte e Nordeste.^(3)^ Na literatura mundial, existe uma tendência de aumento na incidência de doenças fúngicas invasivas, principalmente em pacientes imunodeprimidos. Tanto no Japão como nos Estados Unidos, foi encontrada uma incidência de zigomicose de 2 a 4% nas autópsias de pacientes de centros oncológicos.^(3)^

A zigomicose possui diversas apresentações clínicas (cutânea, pulmonar, sistêmica e gastrintestinal), porém a rino-orbito-cerebral é a mais frequente.^(4, 5)^

Os agentes etiológicos são zigomicetos de duas ordens: *Mucorales*, que acomete principalmente pacientes imunodeficientes e portadores de doenças de base, e *Entomophthorales*, que atinge pessoas imunocompetentes e raramente causa doença angioinvasiva.^(1, 5)^

O diagnóstico está apoiado na correlação entre os exames micológico e histopatológico, e nas manifestações clínicas.^(6)^ A pesquisa do fungo pode ser realizada pelo exame direto com KOH e pela cultura em meio Sabouraud. A análise histopatológica revela a presença de hifas não septadas com ramificações em 90°, geralmente sem invasão de tecidos e de vasos sanguíneos.^(7)^

Os sintomas mais comuns incluem: obstrução nasal, coriza e sinusite crônica,^(8)^ porém a doença pode se manifestar também com febre, letargia, dor de cabeça, dor retro-orbitária, perda abrupta de visão, proptose, celulite periorbitária, epistaxe e convulsão.^(9)^

Ao exame físico, podem ser encontradas crostas necróticas no septo nasal, cornetos e pálato. O envolvimento orbitário pode ser demonstrado, inicialmente, por proptose e celulite periorbitária, com subsequente oftalmoplegia e amaurose.^(6)^ Tardiamente a infecção pode se disseminar para o sistema nervoso central, por meio do etmoide e do seio esfenoidal, após destruição óssea.

O sucesso do tratamento depende da rapidez do diagnóstico^(5)^ e de uma abordagem combinada entre procedimento cirúrgico e terapia antifúngica.^(4)^ A droga de escolha é a anfotericina B. Existem trabalhos que mostram um aumento na sobrevida dos pacientes após a introdução desse tratamento em até 79%. Para controle efetivo da doença, devem-se usar doses elevadas, que variam de 0,8 a 1,5mg/kg/dia − doses muito próximas dos níveis nefrotóxicos.^(9)^

A utilização de fatores de estimulação hematopoiética e oxigenioterapia hiperbárica pode ser benéfica, porém poucos estudos comprovam a eficácia desses procedimentos.^(10)^ Há relatos descrevendo sucesso com a inclusão de iodeto de potássio via oral.^(1, 6)^ O debridamento cirúrgico, o qual tem o objetivo de remover o máximo de tecido desvitalizado possível, além de estabelecer adequada drenagem sinusal, tem grande impacto na morbimortalidade da doença.^(1)^

A prevenção consiste no controle ambiental, evitando ou minimizando o contato direto com propágulos fúngicos presentes em plantas, flores e na poeira doméstica.^(7, 10)^

São fatores de mau prognóstico: atraso no início do tratamento, envolvimento intracraniano (hemiplegia e hemiparesia), palatal ou orbital, assim como envolvimento bilateral de seios da face e a necrose facial.^(7)^

Este trabalho relatou o caso de um paciente imunocompentente, cujo diagnóstico foi tardio e a evolução clínica, inicialmente desfavorável, sendo a maioria dos tratamentos baseada na experiência do médico que o assistia e em relatos de casos encontrados na literatura, resultando em remissão total do quadro, sem nenhuma sequela.

## RELATO DE CASO

Paciente de 9 anos, gênero masculino, procedente de Belém (PA), internado na enfermaria de pediatria do Hospital Israelita Albert Einstein (HIAE), em São Paulo (SP), para tratamento de zigomicose rinofacial. Criança previamente hígida, com o hábito de cheirar rosas. Em março de 2010, apresentou tumoração em região interna da narina esquerda de aumento progressivo. Diagnosticada como celulite, o paciente foi tratado com antibióticos e corticoides tópicos e sistêmicos, sem melhora e até com piora da lesão. Realizada tomografia computadorizada de crânio que evidenciou sinusopatia de seios paranasais esquerdos (Figura 1). Cerca de 3 meses depois, foi submetido à limpeza cirúrgica dos seios faciais com ressecção da lesão de parede nasal. O exame histopatológico evidenciou zigomicose. Duas semanas após, o paciente passou a apresentar celulite acompanhada de dor na região orbitária (Figura 2). Foram iniciadas anfotericina B e caspofungina. A doença continuou a evoluir, apesar das medicações, com piora da dor, diminuição da mobilidade ocular e proptose. O exame de ressonância magnética mostrou invasão periorbitária, com provável contiguidade para a base do crânio (Figura 3). Com esse quadro, a criança foi novamente submetida a duas limpezas cirúrgicas, e o tratamento antifúngico foi prolongado. A partir de então, a evolução passou a ser favorável, com redução das lesões e do edema periorbitário. Na cultura das secreções paranasais, foi isolado o fungo *Conidiobollus sp*.

**Figura 1:** Tomografia computadorizada de seios da face. Sinais de persistência de lesão junto aos limites dos sítios cirúrgicos, na fossa pterigopalatina esquerda, nas cavidades antrais remanescentes à esquerda e de maior componente orbitário também à esquerda, infiltrando planos pré e pós-septais e o aparelho lacrimal

**Figura 2:** Acometimento externo da micose facial

**Figure:** Figura 3 (A e B). Lesão infiltrativa que acomete a face e cavidades paranasais, principalmente na região orbitária esquerda, nariz e orofaringe, com maior acúmulo de secreção nas cavidades paranasais e aumento discreto do edema e/ou processo inflamatório na base da fossa cerebral anterior

Após estabilização do quadro, a criança retornou a Belém para completar o tratamento com anfotericina B. O aparecimento de efeitos adversos da medicação, como hipocalcemia, hipertermia e dores no corpo, resultou em internação em unidade de cuidados intensivos para estabilização hemodinâmica e hidroeletrolítica. Após alta da unidade de terapia intensiva, o tratamento foi substituido pelo antifúngico posaconazol via oral, com deterioração do quadro clínico.

O paciente retornou para o HIAE em agosto de 2010, e um novo exame de ressonância magnética de crânio evidenciou progressão da lesão infiltrativa (Figura 4), acometendo hemiface esquerda, órbitas e maior extensão dos seios etmoidal e esfenoidal, além de acometimento paquimeníngeo. A piora clínica, com a presença de febre persistente e de complicações inerentes à internação hospilar, levaram ao esquema terapêutico com anfotericina B, caspofungina, terbinafina, itraconazol, corticoide, aciclovir, eritropoetina, ceftriaxone, cefepime e ciprofloxacina em tempos diferentes, além de oxigenoterapia hiperbárica. Quinze dias após a última seção da câmera hiperbárica, a ressonância magnética já mostrava lesão localizada sem sinais de invasão de tecidos sãos (Figura 5). Após aproximadamente 2 meses, submeteu-se à nova limpeza cirúrgica, dessa vez com melhora da sintomatologia obstrutiva e praticamente cura da doença.

**Figura 4:** Espessamento e realce envolvendo bulbo e nervos olfatórios e soalho da fossa craniana anterior. Há deslocamento da base do lobo frontal esquerdo, e lesão sugestiva de inflamação e/ou edema nos giros retos e frontais orbitários deste lado

**Figura 5:** Diminuição da lesão infiltrativa que acomete o tecido subcutâneo, principalmente periorbitário esquerdo, e redução da proptose do mesmo lado. Redução da lesão na rinofaringe, hipofaringe e seios da face, em comparação com os anteriores

## DISCUSSÃO

*Conidiobollus sp* é um saprófita do solo e seus propágulos estão presentes na vegetação em geral. Pode parasitar artrópodes, sendo a infecção humana acidental.^(10)^ A contaminação com o fungo se dá por via inalatória ou inoculação direta, como no caso do paciente descrito.^(8)^

O quadro clínico inicial, com lesão nasal de crescimento rápido provocando sinais obstrutivos que, em poucas semanas, difunde-se para a região periorbitária, causando edema, ptose e proptose, é a apresentação mais comum da doença.^(1)^

A demora de cerca de 3 meses até o diagnóstico definitivo justifica a alta morbidade da doença e pode ser explicada pelo grande leque de diagnósticos diferenciais para os sintomas iniciais.^(2)^ A lesão de pele foi inicialmente tratada como impetigo e os sintomas obstrutivos foram tratados como sinusite. A ausência de resposta terapêutica resultou na limpeza cirúrgica e na biópsia.^(3)^

É importante considerar que a anfotericina B por via intravenosa tem sido apontada como tratamento de escolha.^(1, 6, 7)^ No paciente em questão, esse fato ficou bem documentado, uma vez que, nos períodos nos quais ele recebeu essa droga pela via adequada, a resposta foi mais satisfatória. No entanto, para que o tratamento possa ser feito de modo completo, são indispensáveis os cuidados com a administração do medicamento, a fim de evitar os efeitos adversos também observados nesta criança.

A oxigenoterapia hiperbárica pode ter contribuido para a melhora, embora não existam evidências consistentes sobre seu emprego nessa indicação.^(10)^ Finalmente, a limpeza cirúrgica é parte essencial da estratégia terapêutica, já que retira o máximo de material infectado possível e permite viabilizar a drenagem sinusal, como observado no caso, devendo seu emprego ser enfatizado.^(7)^

## CONSIDERAÇÕES FINAIS

A zigomicose é uma doença fúngica grave, que pode acometer tanto pessoas imunocompetentes como aqueles com a imunidade comprometida.

A literatura mostra que a rapidez no diagnóstico e o início precoce do tratamento adequado são fundamentais para o prognóstico da doença. Porém, o paciente descrito no caso, embora tenha tido um atraso no diagnóstico, evoluiu de forma favorável, devido às máximas terapêuticas empregadas.
